# Miscellanies

**Published:** 1828-01-01

**Authors:** 


### Miscellanies.

### FACETIÆ LITERARUM MEDICARUM.

### I.

" HOW TO BECOME A POPULAR EDITOR OF A MEDICAL MISCELLANY.

[*From the* Dissector, *No.* 2.]

" Begin by publishing an account of a beastly crime—rob other men of their intellectual property to fill your pages—praise the scum of the profession to the skies, and assail honest and respectable men with nick-names, the lowest ribaldry, and wretched wit. By so doing you will rise from obscurity into disgraceful *notoriety*—the little lecturers will shake your hand, admit you at their houses, and supply you with a corrected copy of their lectures. Thus, you will insure the sale of your miscellany, and gain a threadbare living, which you might never have done in practice."

### II.

WANTED FOR A MEDICAL MISCELLANY.

[*Ibidem.*]

1. " Anecdotes of private or professional character, with just sufficient truth to give a plausible covering to the falsehoods which it may be expedient to found upon them.

2. " Hospital reports—no matter if they proceed from empty walls.

3. " Pithy answers to imaginary correspondents.*

4. " A translator of the German and Italian. It will not be required that he should be conversant with either language.

5. " A set of well-rounded encomiastic periods for friends, together with a few nick-names for opponents.

6. " The use of Juvenal's Satires, with the *English under each word,* for occasional quotation.

7. " A Lancet *unsoiled* by malignant matter,—for shew only."—Dissector, No. 3.

* We beg to supply the Dissector with an example.

" We have just received a communication of 50 foolscap pages from our correspondent in the Moon. We shall return a more particular answer by the *Sky-rocket* packet, which starts from our office every Friday, at 2 o'clock, precisely."

### III.

### CONSISTENCY.

| LANCET. | LANCET. |
|---|---|
| 1. " Some who *cant*—others who *recant*."—*Vol.* III. *p.* 18. | 1. " If a man be desirous of plugging up a tortuous passage, he does not select a piece of *unyielding* oak"—*viz.* Lawrence !—*Vol.* XIII. *p.* 20. |
| 2. " Neither the fracture of the cranium nor that of the femur had been discovered during life; so much for Bartholomew's surgery. The patient was under the care of Mr. Lawrence."—*Ib.* III. *p.* 190. | 2. " The publication of hospital reports has shewn the public the spot on which the genius of Lawrence shines. Pupils are now *flocking* from all parts of the kingdom eager to be illuminated."—*Vol.* XII. *p.* 620. |
| 3. " We have, in the lectures of Mr. Abernethy, matter which will be found highly amusing and instructive."—*Vol.* II. *p.* 417. | 3. " The lectures which we now present to our readers complete Mr. Abernethy's surgical course; and as we are of opinion that they form no embellishment to our pages, we have thought it best to shake off the INCUBUS at once."—*Vol.* VII. *p.* 1. |
| 4. " Mr. Green is evidently very vain and a very shallow person."—*Vol.* III. *p.* 371. | 4. " Mr. Green has honourably distinguished himself from most other hospital teachers."—*Vol.* XIII. *p.* 99. DISSECTOR, No. 4. |
| MR. BELL.—" We should lament giving to the public any imperfect performances of this perfect surgeon."—*Vol.* V. *p.* 13. | " Here, Gentlemen, (holding in his hand an empty skull,) I compare this to a BELL, and I am sure you will at once perceive the aptitude of the simile."—*Vol.* IX. *p.* 878. |
| DR. BIRKBECK.—" Dr. Birkbeck has deserved well of his country—he is a real friend of the human race "—*Vol.* XI. *p.* 845. | " Old Brickbat, the star of the East, so radiant he shines on mechanics."—*Vol.* X. *p.* 29. |

### NO PERSONALITIES.

| | EXAMPLES. |
|---|---|
| " When and where have we noticed any official character in a way which can justly be called *personal?*" DISSECTOR, No. 8. | "Ninnyhammers"—"Toads"—"Bats"—" Hospital Idiots"—" Joe Green"—" Joe Burns"—" The Surgical Eclipse"—"The *Huge* Eclipse"—" Ben Travers"—"Little Brodie"—" Cockney Mayo"—" Saint Julius"—" Bandager Jeffrey"—" The Prickley Rose"—" Sir Everard Fuddle"—" Dowager Lynn"—"The Yellow Goth," &c. &c. Passim. |

---

### IV.

### RETROSPECTIVE VIEWS OF A PLEASING KIND.

[*From the first number of the London Medical Gazette.*]

" At no distant period, the profession of medicine was a respectable, peaceful, and comparatively happy pursuit; none of its members were exposed to attacks from the press, but those who invited them by the act of publication; the rest followed their occupations in privacy, with no other interruption to their tranquillity than the toils and anxieties of their profession. To industrious and conscientious men, these were enough, and often more than enough—and many a one broke down under them.

"But a few years ago, a set of literary plunderers broke in on the peace and quiet of our profession. Lecturers, who had spent their lives in collecting knowledge, arranging it for communication, and acquiring the difficult art of oral instruction, saw the produce of their lives suddenly snatched from them, and published for the profit of others, with the additional mortification of finding what they had taken so much pains with, disfigured by bad English and ridiculous or mischievous blunders. Whoever attempted to arrest these piracies became the object of furious and unrelenting abuse. Hospital physicians and surgeons, who have to prescribe and operate in public, and at stated times, in whatever condition of bodily health or mental feeling they may happen to be, and exercising in the face of critics not always competent to decide on their merits,—a science so avowedly imperfect, as to afford abundant scope for uncandid and ill-natured remarks, however judiciously practised,—were held up to public scorn for errors, to which, even if actually committed, the ablest men are occasionally liable, while those who are leagued in secret with their calumniators, and who, with one or two exceptions, were as insignificant in station and talents as they were equivocal in character, were represented as at the summit of science and professional eminence.

"Such, among others, have been the results of a system, which has no parallel in the records of any liberal profession. We do not deny that public exposure may have, in some few instances, done good; it may have abolished some foolish custom, or led to the reformation of some trifling abuse; but weigh the evil against the good—it has deprived eminent men of their intellectual property, and destroyed the mutual confidence between pupils and their teachers; it has lowered the respectability of the profession, and has spread general distrust; it has broken up private friendships; it has placed man in hostility to man; and has set so many bad passions into ferment, that well-disposed men become disgusted with the state of their profession, and vow that they never will inflict it on their sons."

### V.

### DR. HOPKINS' PRIZE MEDAL.

We are at all times happy to bear testimony to the efforts of medical professors in forwarding the views and welfare of their pupils. Amongst the foremost of these, we may remark the great exertions used by Dr. Hopkins, to increase the means, and rouse into activity the energies, of his pupils, to acquire a thorough knowledge of that most important branch of medical study, the science and practice of midwifery.

Now, we conceive the methods Dr. H. has adopted to effect these desirable purposes, cannot fail of meeting with that approbation and success which his liberality alone, for we can call it by no lesser mark of distinction, as will presently appear, should entitle him to expect. Dr. H. had contemplated, for some time past, on the plan of distributing prizes to his class; and accordingly, with this intention, Dr. H. has invented an appropriate and elegant design, from which a *gold medal* has been cast, and is now held out, as a prize, to his pupils at the Borough School, for the best Essay on the Various Causes and Treatment of Protracted Labour: and likewise to his pupils at the West End School, for the best Essay on Complex Labour.

We may further observe, that, with the commendable anxiety to excite the utmost exertions of his pupils, and in consideration of a number of well written essays having been sent in, the Doctor has been induced to offer a silver medal (the same design as the gold one) for the second best essay.

We are given to understand, besides fostering the most laudable feelings of emulation amongst his classes, by conferring these handsome and valuable prizes, the Doctor is constantly assisting and improving their studies, by attending patients with them at the bedside, and making those useful and practical remarks which he considers applicable to each individual case.

Society at large will at once acknowledge, such a man not only deserves well of his brethren in the profession, but also of every philanthropist and well-wisher of mankind, in thus endeavouring to further this most important pursuit.

